# Infant Drowning Prevention: Insights from a New Ecological Psychology Approach

**DOI:** 10.3390/ijerph19084567

**Published:** 2022-04-11

**Authors:** Carolina Burnay, David I. Anderson, Chris Button, Rita Cordovil, Amy E. Peden

**Affiliations:** 1 School of Physical Education, Sport and Exercise Sciences, University of Otago, Dunedin 9016, New Zealand; chris.button@otago.ac.nz; 2 Marian Wright Edelman Institute, San Francisco State University, San Francisco, CA 94132, USA; danders@sfsu.edu; 3 CIPER, Faculdade de Motricidade Humana, Universidade de Lisboa, 1495-751 Cruz Quebrada, Portugal; cordovil.rita@gmail.com; 4 School of Population Health, Faculty of Medicine and Health, UNSW Sydney, Kensington, NSW 2052, Australia; a.peden@unsw.edu.au; 5 College of Public Health, Medical and Veterinary Sciences, James Cook University, Townsville, QLD 4811, Australia

**Keywords:** epidemiology, water safety, affordances, water cliff, water slope, child, development, environment, risk

## Abstract

Drowning causes significant mortality and morbidity globally, and infants (0–4 years of age) are disproportionately impacted. In a groundbreaking approach to pediatric drowning prevention, ecological psychology has been used to investigate the relationship between infants’ perceptual–motor development and their behavior around bodies of water. In this review, we summarize recent research findings in the field of ecological psychology and apply these to the prevention of infant drowning. Studies have linked infants’ avoidance of falls into the water with locomotor experience and type of accessway into bodies of water. Through crawling experience, infants learn to perceive the risk of falling into water and start adapting their behavior to avoid drop-offs leading into water. Infants tend to enter deep water more when the access is via a slope than via a drop-off. We propose that ecological psychology can enhance infant drowning prevention interventions. The aim is to create an additional layer of protection, the perceptual information layer, in addition to existing strategies, such as supervision and barriers. This new protective layer can be a powerful tool to further highlight the risk of entering the water and reduce infant drowning-related mortality and morbidity.

## 1. Introduction

Drowning has been identified as a public health threat by the World Health Organization (WHO) [1]. The United Nations (UN) General Assembly [2] resolution suggests drowning prevention strategies must be prioritized by governments. Over half of all fatal unintentional drownings globally occur among children and young people under 25 years of age, with children 0–4 years of age recording the highest drowning rates [1]. Drowning often occurs among this age group in water bodies inside the home for children under 1 (such as bathtubs) [2] and in water bodies outside but close to the home (such as ponds, ditches, swimming pools), in children 1–4 years of age [3]. Drowning among young children is due to a range of age-related developmental factors and hazards and risks in the child’s environment (e.g., unrestricted access to water, lapses in or absence of adult supervision) [4].

The first few years of human life are characterized by an extraordinary number of developmental changes that occur at a remarkably rapid pace. Infants younger than 12 months of age are dependent on their caregivers to survive; they are fed, cleaned and, before they start self-locomoting, they are carried. Infants grow every day, their body dimensions and capabilities change, they conquer new skills, they gradually become more independent, and they are eager to explore the world. Although this exploratory behavior is vital for infants to learn to distinguish possible from impossible (or dangerous) actions [5], it also puts them at risk of injuries from failures to accurately perceive risks in the environment.

## 2. What Do We Know about Infant Drowning Risk?

Epidemiological data have been used to develop strategies to prevent drowning [6]. This approach is essential to understand who is at risk and where drowning is more likely to occur. Importantly, by having updated epidemiological information, we can monitor the effectiveness of drowning prevention strategies.

The Global Burden of Disease (GBD) Study estimates that children 1–4 years of age have the highest number of unintentional drowning deaths when compared to other age groups [7]. In 2019, drowning resulted in 32,070 deaths (95% uncertainty interval (UI): 26,399.60–39,587.44) of children 1–4 years of age, an age-adjusted fatality rate of 6.04 (UI: 4.97–7.46) per 100,000 children [8]. Drowning also results in 1469.91 (UI: 975.50–2108.07) years lived with disability (YLDs) among children in the 1–4 years age group [8]. Among infants younger than 1 year of age, unintentional drowning claims the lives of an estimated 867 children (UI: 619.93–1089.83), a mortality rate of 2.64 per 100,000 children [8].

The highest fatal drowning rates are seen in low socio-demographic index (SDI) countries, a rate of 3.99 per 100,000 for children less than 1 year of age and 8.55 per 100,000 for 1–4-year-old children in low SDI countries, compared to a rate of 0.77 for children less than 1 year of age and 1.40 for 1–4-year-old children in high SDI countries [8]. The high proportion of drownings with a fatal outcome in low SDI nations is evident in the lower YLD-related drowning rates for children less than 4 years of age, when compared to those from high SDI nations [8].

## 3. The Multiple Layers of Protection

Drowning prevention is most effective when multiple layers of protection work together [6]. Infants are overrepresented in drowning statistics because they become “mobile but [are] too young to recognize danger or to get out of water” (p. 9) [1].

To protect mobile infants from drowning, the WHO suggests four strategies that constitute layers of defense [1]. The first and most obvious suggested layers of protection are barriers around water, such as pool fencing [9,10] and adult supervision [1,6]. However, barriers are not always an option, nor absolutely childproof [11]. Some bodies of water, such as beaches or rivers, cannot be fenced [12]. In addition, research has shown that no barrier can assure a 100% effective means to prevent infants from getting to the water [13]. If the body of water is not fenced or if children manage to overcome the protective physical barriers, adult supervision is the next most effective protective layer. However, as shown by Moran [14], it is not uncommon that children find themselves close to a body of water with limited or no adult supervision. Similarly, young children may be left in the care of older children, unsuited for the provision of supervision required to reduce drowning risk [15,16], or even distracted adults [15].

Swimming skills are suggested as the third layer that ultimately can help children to survive in the water. However, the benefits of swimming programs to very young children (younger than three years of age) have been historically controversial. In the 1970s and 1980s, experts, including the American Academy of Pediatrics, discouraged swimming programs for children younger than three years, arguing that this kind of exposure would offer a false sense of security to the children and their parents [17,18]. Recently, a systematic review conducted by Taylor and colleagues reported that children aged 2–4 years can develop age-appropriate aquatic competencies, and the learning of these skills may increase water safety [19]. However, a very limited number of studies have addressed the effect of swimming ability on preventing drowning for young children, and no evidence was found that children under 1 year of age are capable of learning how to survive in the water because they cannot intentionally control their breathing [19]. Therefore, the benefits of swimming courses for infant drowning prevention need to be further investigated [19].

It seems then that if barriers and adult supervision fail, young children are completely vulnerable to drowning. While devices such as lifejackets play an important role in drowning prevention [20], their appropriateness for young children and the typical drowning scenarios seen among this age group are questionable. This leaves a final and least reliable layer of defense, i.e., rescue and resuscitation [21].

As stated by the WHO, although the epidemiological approach has largely contributed to the development of important strategies to prevent drowning, they are not enough to capture drowning risk factors and effectiveness of interventions alone. There is a need for new research approaches to further our understanding of the mechanisms behind these fatal yet preventable incidents [1]. This review describes an ecological psychology approach to drowning prevention for infants. We define infants as comprising the 0–4 years age group, but we cluster our discussion of the literature into those under 12 months of age and those aged 1–4 years, due to differences in locomotor capabilities/skills and drowning risk. We propose that stronger linkages between the epidemiological and ecological fields of research are required for the development of potentially innovative interventions for these at-risk groups.

## 4. The Ecological Psychology Approach to Infant Drowning Prevention

Ecological psychology focuses specifically on the interdependence of humans and their environments. It stresses that perception is an active process and that action and mobility play particularly important roles in modulating perceptual processes and their development [22]. How children perceive possibilities for action, or affordances [23], depends on the relationship between their characteristics, including the interaction of various developing systems (e.g., perceptual, motor, cognitive, etc.), and the characteristics of the environment. Through the use of their own bodies during goal-directed activity, children get acquainted with their surroundings and begin perceiving affordances in the environment [22]. When infants start self-locomoting, a solid surface affords crawling or walking, but the water surface does not. Although infants’ capacity to control locomotion improves rapidly, their perception of the affordances for locomotion lags behind their capacity for control. They need to perceive the relationship between their capabilities and the environment’s features to avoid drowning.

Ecological psychology has long been used to investigate child–environment relationships and the effects of perceptual–motor development on infants’ behavior in risky scenarios, such as drop-offs [24], but only recently have researchers begun to investigate infants’ relationship with bodies of water from an ecological perspective.

This ecological line of investigation started with the classical *Visual Cliff* paradigm [25]. Eleanor Gibson and Richard Walk (1960) tested the avoidance behavior of human babies, among other animal species, on a drop-off covered with a transparent glass surface, the visual cliff [25]. They reported that crawling infants would avoid crossing visual cliffs, similar to other young animals [25]. However, Gibson and Walk did not compare infants at different stages of locomotor development [25].

Later studies using the classical visual cliff paradigm and its adaptations have shown that soon after infants start crawling, they tend to cross visual cliffs [26] and risky slopes [27] and to fall into gaps in the surface [28] and even over the edge of real cliffs [29]. After weeks of crawling experience, infants start perceiving the risk of falls and start adapting their behavior to avoid visual cliffs [30], steep slopes [31], impossible-to-transverse gaps [28] and dangerous drop-offs [29].

**Figure 1:**
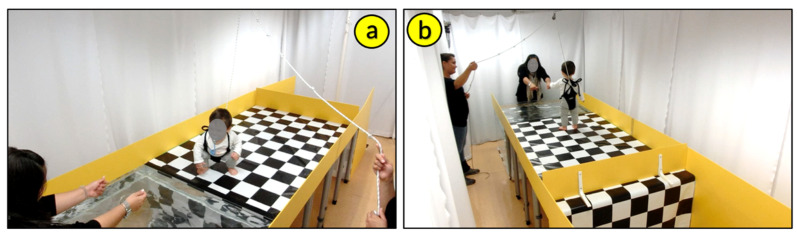
*Real Cliff/Water Cliff* apparatus synchronized cameras’ view. (**a**) water cliff camera front view and (**b**) water cliff camera back view. Photo reproduced with permission of the infant’s mother.

In a literally “ground-breaking” approach, ecological psychology and the related concept of affordances was used to develop a novel approach to infants’ drowning prevention. To investigate the interaction between infants’ perceptual–motor development and aquatic environments, Burnay and Cordovil [32] adapted the visual cliff paradigm and created the *Real Cliff/Water Cliff* apparatus (i.e., 75-cm-high platform with no protection from a fall on one side, the real cliff, and one with a tub filled with water on the opposite side, the water cliff) (Figure 1). Burnay and colleagues tested 58 crawling and 44 walking infants [32,33] and 25 infants who transitioned from crawling to walking in a longitudinal study design [34] on both the real and water cliffs. The results confirmed the effect of crawling experience on infants avoidance of real cliffs and showed the same effect on infants avoidance of the water cliff [32,33,34]. Infants with more crawling experience avoided falling on the real and the water cliffs and their behavior was indiscriminable on the real and the water cliff. For the first time, locomotor experience was linked to infants’ avoidance of bodies of water.

Through crawling, infants learn to perceive information specifying important characteristics of the surface of support and start avoiding situations that do not support safe locomotion. After acquiring the necessary self-produced locomotor experience, when facing a drop-off, filled with water or not, babies avoid going farther because they perceive the environment does not afford safe locomotion.

These studies raised a new question: what if, instead of a sudden drop-off, the access into the water is smooth and gradual? In many natural aquatic environments, such as beaches and ponds, and in swimming pools designed to facilitate entry for people with disabilities, entrances to the water can be less obvious than a sudden drop-off. Would self-produced locomotor experience inform infants’ perception of the risk when slopes lead into deep water?

**Figure 2:**
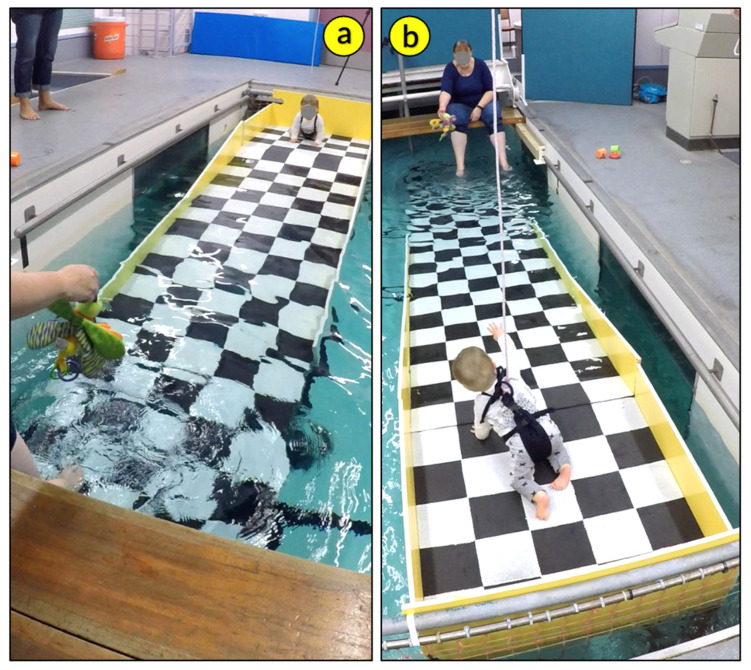
*Water slope* apparatus synchronized cameras’ view. (**a**) camera front view and (**b**) camera back view. Photo reproduced with permission of the infant’s mother.

To answer this question, in a follow-up study, Burnay and colleagues [35] created the *Water Slope* paradigm to test infants’ perception and action on sloped accessways into deep water. The water slope is a 10° declined platform leading to 75-cm-deep water (Figure 2). Contrary to what was observed on the water cliff, self-produced locomotor experience was not linked to infants’ avoidance of submersion on the water slope (i.e., water touching the infants chin) [35]. Importantly, of the 77 infants tested on the water slope, 62% reached the submersion point [35], while on the water cliff, of the 102 infants tested on a cross-sectional design [35], only 30% fell into the water. The authors argue that if the access into the water affords locomotion (i.e., smooth slope), infants tend to locomote, presumably because locomotor experience has no influence on infants’ perception of the risk of deep water. Hence, it seems that a sloped accessway to water may increase the chances of infants engaging in drowning incidents. However, infants from Portugal were tested on the water cliff, and infants from New Zealand were tested on the water slope. Whether the greater tendency the infants showed to engage in dangerous behavior on the slope when compared with the drop-off is only the result of the different perceptual information offered or whether cultural differences between the two countries had a role in infants’ behavior needs to be further investigated. Another limitation of the water cliff and water slope study paradigms is that they were conducted in laboratory environments. If we want to understand how infants would behave in the real world, these studies should be replicated in more ecological environments.

## 5. Gaps in Our Understanding and Directions for Future Research

As shown by Burnay and colleagues, when infants start crawling, they tend to fall over drop-offs leading into the water [32,33,34]. Infants need to acquire enough self-locomotor experience to start perceiving the risk and avoiding falling into the water. The acquisition of self-locomotor capability and the gap between the time infants first start locomoting until they have sufficient experience to act adaptively according to the features of the environment is a period when infants are at increased risk of falling into bodies of water. However, by the time infants acquire sufficient self-locomotor experience (i.e., between 1 and 2 years of age, sometimes earlier), they should be capable of perceiving the risks of falling into the water and avoid it. Yet, infants 2–4 years of age are included in the 1–4 years-of-age overrepresented group of children at greatest risk of drowning [3,4,7].

Statistically, in high-income countries, before becoming mobile (under 1 year of age,) babies drown in bathtubs and water inside the home. Between the ages of 1 and 4, when babies have acquired the capability to locomote, drownings occur mostly in bodies of water around the home, such as private swimming pools, ponds and dams [3,10,15]. The ecological studies on infants’ behavior in response to different types of access into the water have shown an increased tendency for mobile babies to enter deep water when the access is smooth and gradual. However, epidemiological data do not typically reference the design of the accessway into the water (i.e., sloped or sudden drop-off).

## 6. Future Directions to Eradicate Infant Drowning

Current and previous research on drowning is epidemiological in nature [36]. It describes rates of drowning and highlights who is at most risk of drowning and in what contexts. This research has led to the development of drowning prevention strategies focused on fencing swimming pools and ensuring young children are closely supervised around bodies of water at all times. Yet, high drowning rates among infants persist.

### 6.1. Manipulation of the Aquatic Context: Building a New “Perceptual Layer of Protection”

The pioneering ecological psychology approach to studying infants interaction with aquatic environments has proven to be an important means to investigate why infants drown, what developmental variables are linked to their adaptive behavior and how their behavior changes when the aquatic context changes. Importantly, prior studies utilizing this approach have shown that we can modify the environment, specifically the perceptual information offered by the context around and leading into the water, to make infants behave more adaptively [35]. In this section, we provide some specific examples of how aquatic environments can be designed to further reduce the risk of infants unintentionally falling into water unsupervised. By manipulating the perceptual information infants receive when approaching a body of water to increase their perception of the risk, we can create a new layer of defense from drowning: the perceptual layer.

Future research should further investigate infants’ perception of risk and consequent behavior in different aquatic contexts. For instance, visible texture on the supporting surface has been demonstrated to play a role in infants’ tendency to go over drop-offs. Eleanor Gibson and colleagues [37] compared infants’ avoidance of a visual cliff when a matte black surface or a surface with visible texture were presented and reported a greater tendency of infants to cross the visual cliff in the visually textured condition. Should visually textured surfaces be avoided when designing the surrounds of aquatic environments, such as public and private swimming pools?

Previous research has shown that the visual information on the vertical surroundings can impact infants’ avoidance of drop-offs. Anderson and colleagues [38] reported that infants’ crossing probability on the visual cliff increased when the walls surrounding the apparatus were high textured (i.e., with visual patterns) compared to when the walls where low textured. Should fences surrounding bodies of water be placed far away from the water? Or, better yet, should such barriers be made transparent? Studies are urgently needed to address these questions.

Other environment design features can also be investigated using the ecological framework. For instance, would painting contrasts between the dry and wet sections of a sloped entrance into a pool enhance infants’ perception of the risk of going further into the water? In addition, even though perceptual psychology has been biased toward studying visual perception, paying little attention to other senses, James J. Gibson [39] emphasized that a functionally active person will see, hear, touch and taste to interact adaptively with his/her environment. Therefore, design considerations related to infants’ drowning prevention should not be restricted to visual information alone. The role of multiple sources of perceptual information on infants’ perception of affordances needs to be investigated. Perhaps another way to make water access points more obvious to infants, for example, is to include design stones or an uneven or roughly textured surface to mark the boundary between dry and wet ground. Insights from studies such as these, underpinned by ecological psychology, can inform swimming pool design guidelines and policy regulations to create a new enriched “perceptual layer of defense” for pediatric drowning prevention.

### 6.2. Manipulation of Infants’ Aquatic Experiences to Improve Their Perception of Risk

As previously established, teaching aquatic skills may not be an effective means to prevent infants from drowning because infants younger than two years of age cannot acquire appropriate aquatic skills [19]. However, would exposure to aquatic environments enhance infants’ perception of the risk of entering the water? Or would this exposure give them a false sense of security leading to a less adaptive behavior? The ecological approach can be used to investigate if different methodologies for teaching swimming have different effects on infants’ perception of the risk of entering the water. Interventions in which infants are exposed to different aquatic experiences (e.g., swimming classes, free playful exploration) with pre- and post-tests of their avoidance of bodies of water would provide important information about the best aquatic activities for enhancing infant water safety.

Another issue the ecological psychology approach can help resolve is the disparity in drowning statistics among racial/ethnic minority groups [40]. In the United States, African American children have the highest drowning fatality rates, followed by American Indian and/or Alaskan natives, Whites, Asian American and/or Pacific Islanders and Hispanic children [41]. The WHO suggests differences in swimming ability and experience in the water, including opportunities to learn to swim, may contribute to disparities in risk of drowning among racial/ethnic groups [41]. However, they also recognize that these contributing factors are speculative, poorly understood and require further investigation [1]. The ecological approach can be used to investigate whether different racial/ethnic groups have different relationships with aquatic environments, whether they perceive the risk imposed by bodies of water differently or whether disparities in drowning are a matter of different opportunities to explore the water and to learn swimming skills.

Button et al. [42] describe how the ecological approach can help practitioners in less developed countries to appreciate how the individual–environment relationship impacts water safety skills transfer and thereby reduce the risk of drowning. This is important, as drowning is particularly prevalent in low- and middle-income countries due to the lack of resourcing for controlling access to open water and limited water safety education.

## 7. Conclusions

A closer alignment between the fields of drowning epidemiology and ecological psychology should be paramount to those interested in behavioral interventions to prevent pediatric drowning. The ecological psychology approach shows that the more crawling experience infants have, the more adaptively they behave in risky situations, and that sloped entrances to the water may increase infant drowning risk. However, epidemiologic studies rarely refer to infant developmental stages [4], nor to the type of accessway into the water when reporting pediatric drowning statistics.

A promising research direction is to consider how differently designed aquatic entranceways and different aquatic experiences can provide an additional perceptual layer of defense to help protect those most vulnerable to drowning. It is also clear more work is needed to transfer the findings from studies highlighted in this review to low- and middle-income contexts, where drowning is less common in built environments, such as home swimming pools.

Collaboration between the fields of drowning epidemiology and ecological psychology can inform innovative strategies that can address the tragedy of pediatric drowning and thereby keep babies safer around water.

## Data Availability

Not applicable.
